# Pulse oximetry for the prediction of acute mountain sickness: A systematic review

**DOI:** 10.1113/EP091875

**Published:** 2024-09-25

**Authors:** Johnathan S. L. Goves, Kelsey E. Joyce, Sophie Broughton, Julian Greig, Kimberly Ashdown, Arthur R. Bradwell, Samuel J. E. Lucas

**Affiliations:** ^1^ Anaesthesia & Intensive Care Medicine Royal Blackburn Teaching Hospital, NHS Trust, Haslingden Road Blackburn UK; ^2^ School of Sport, Exercise and Rehabilitation Sciences University of Birmingham, Edgbaston Birmingham UK; ^3^ Birmingham Medical Research Expeditionary Society University of Birmingham Birmingham UK; ^4^ Medical School University of Birmingham Birmingham UK; ^5^ Occupational Performance Research Group University of Chichester, College Lane Chichester UK

**Keywords:** acute mountain sickness, high‐altitude, pulse oximetry

## Abstract

Acute mountain sickness (AMS) causes serious illness for many individuals ascending to high altitude (HA), although preventable with appropriate acclimatisation. AMS is a clinical diagnosis, with symptom severity evaluated using the Lake Louise Score (LLS). Reliable methods of predicting which individuals will develop AMS have not been developed. This systematic review evaluates whether a predictive relationship exists between oxygen saturation and subsequent development of AMS. PubMed, PubMed Central, MEDLINE, Semantic Scholar, Cochrane Library, University of Birmingham Library and clinicaltrials.gov databases were systematically searched from inception to 15 June 2023. Human studies involving collection of peripheral blood oxygen saturation ($S_{pO_{2}}$) from healthy lowlanders during ascent to HA that evaluated any relationship between $S_{pO_{2}}$ and AMS severity were considered for eligibility. Risk of bias was assessed using a modified Newcastle–Ottawa Tool for cohort studies (PROPSPERO CRD42023423542). Seven of 980 total identified studies were ultimately included for data extraction. These studies evaluated $S_{pO_{2}}$ and AMS (via LLS) in 1406 individuals during ascent to HA (3952–6300 m). Risk of bias was ‘low’ for six and ‘moderate’ for one of the included studies. Ascent profiles and $S_{pO_{2}}$ measurement methodology varied widely, as did the statistical methods for AMS prediction. Decreasing oxygen saturation measured with pulse oximetry during ascent shows a positive predictive relationship for individuals who develop AMS. Studies have high heterogeneity in ascent profile and oximetry measurement protocols. Further studies with homogeneous methodology are required to enable statistical analysis for more definitive evaluation of AMS predictability by pulse oximetry.

## Abstract (highlights)

- **What is the central question of this study?**
  Is there a predictive relationship between oxygen saturation and subsequent development of AMS?
- **What is the main finding and its importance?**
  A systematic review of the literature revealed there is a positive predictive trend between pulse oximetry measurements obtained around 3500 m during ascent and the development of acute mountain sickness at higher camps; however, further research is required to develop more robust prediction models.

## 1 INTRODUCTION

Acute mountain sickness (AMS) is one of three major high‐altitude (HA, >2500 m) illnesses (including HA cerebral and pulmonary oedema; HACE and HAPE) (Imray et al., 2011) and can afflict as many as 75% of people who ascend to HA (Croughs et al., 2014; Karinen et al., 2008). AMS has a much higher incidence and occurs at much lower altitudes than the more severe syndromes of HACE and HAPE. HA illnesses are caused by exposure to the reduced atmospheric pressure and reduced partial pressure of oxygen relative to sea level, which ultimately creates a hypoxic state in exposed individuals.

AMS and HACE are caused by cerebral oedema due to increased fluid permeability of the blood–brain barrier. The mechanism for how this occurs is unclear but is thought to be multifactorial. Hypoxia, hypercapnia, increased vascular pressures and inflammatory processes have all been linked as vasogenic causes, with other cytotoxic causes also identified (Lafuente et al., 2016). Nevertheless, the cerebral oedema results in the classical collection of symptoms including HA headache, nausea/vomiting, dizziness and fatigue (Luks et al., 2017), and at extreme altitudes these oedematous changes can become profound enough to cause acute central neurological deficits. This is considered the threshold for the diagnosis of HACE.

The gold standard for both AMS and HACE prophylaxis is adequate physiological acclimatisation to HA, which can be achieved through rest periods, and slow and partial ascents (<500 m gain in sleeping altitude per day above 3000 m) (Imray et al., 2015). However, adequate acclimatisation is time consuming, prompting poor adherence and the widespread use of pharmaceuticals to aid the process. Most noteworthy of these prophylactic aids is acetazolamide, a carbonic anhydrase inhibitor that induces mild acidaemia, which stimulates increased respiratory drive, and thereby increases oxygen delivery, thus accelerating acclimatisation (Leaf & Goldfarb, 2007). As such, acetazolamide can also be used for treatment, notwithstanding that both AMS and HACE can be treated with oxygen and immediate descent in severe cases. AMS can also be treated with paracetamol and adequate oral hydration, whereas HACE requires treatment with potent corticosteroids such as dexamethasone to reduce cerebral oedema, thus emphasising the importance of prevention and close monitoring (Joyce et al., 2018).

Currently, diagnosis of AMS remains clinical, and when symptoms are severe, the condition makes diagnosis obvious. Despite this, in its earlier stages AMS can be ill‐defined without distinctive signs or symptoms. The self‐report Lake Louise Score (LLS) criteria can also be used to evaluate AMS, and involves subjectively ranking symptoms, such as headache, gastrointestinal distress, fatigue and dizziness/light‐headedness (Roach et al., 2018). While the LLS is able to track progression of the illness as symptoms develop, and can be used as a diagnostic aid, it currently offers no predictive value (Moore et al., 2020). The subjectivity and disputed reliability of the LLS have emphasised the need for assessing AMS with improved diagnostic accuracy utilising more objective and ideally prospective methodology, which would allow clinicians to identify individuals who are at risk in time for preventative intervention.

Physiological parameters have been investigated to ascertain whether they can be used to form a more objective means of predicting AMS in individuals, as well as assessing severity and susceptibility. Given that AMS is the product of exposure to prolonged hypobaric hypoxia, arterial oxygenation has been the reflexive physiological parameter to investigate alongside AMS. Whilst direct measurement of arterial oxygenation ($S_{{\mathrm{aO}}_{2}}$) requires specialised equipment and techniques that are prohibitively impractical outside of research settings, measurement of peripheral blood oxygen saturation ($S_{pO_{2}}$) via pulse oximetry is more convenient, and more practical for use in the field at altitude. Nevertheless, the literature surrounding the utility of $S_{pO_{2}}$ in evaluating AMS severity appears inconclusive (Major et al., 2012; O'Connor et al., 2004), and the utility of $S_{pO_{2}}$ in AMS prediction even less thoroughly researched.

The purpose of this systematic review is to evaluate the literature related to the use of pulse oximetry at high altitude as a predictive factor for AMS susceptibility and severity, so that individuals likely to develop the condition can be identified early, managed more appropriately, and disease burden on individuals, teams and local resources reduced.

## 2 METHODS

This systematic review is reported in accordance with the Preferred Reporting Items for Systematic Reviews and Meta‐Analyses (PRISMA) guidelines (Page et al., 2021). The protocol for this review was prospectively registered with the international prospective register of systematic reviews (PROSPERO) database (ID: CRD42023423542) (Joyce et al., 2023).

### 2.1 Ethical approval

This systematic review only contains papers that were conducted in line with the relevant version of the *Declaration of Helsinki* and had relevant ethical approval(s) in place. A summary of this information is provided in Appendix C.

### 2.2 Eligibility criteria

Human studies involving the collection of peripheral blood oxygen saturation ($S_{pO_{2}}$, via pulse oximetry) from healthy lowlanders during ascent to terrestrial high altitude that evaluated the relationship between $S_{pO_{2}}$ and AMS severity were considered for eligibility. Studies were excluded that: (1) included animals, unhealthy humans (e.g., known pre‐existing cardiac/metabolic/respiratory condition(s), smokers), or only highlanders (living above 2000 m); (2) utilised simulated altitude (e.g., normobaric/hypobaric hypoxia in an environmental chamber); (3) failed to report the AMS assessment method (e.g., LLS or Environmental Screening Questionnaire, ESQ); (4) measured blood oxygen saturation only by arterial samples; (5) assessed only chronic mountain sickness; (6) measured $S_{pO_{2}}$ only at a single altitude or a maximum altitude ≤2100 m, or (7) failed to evaluate, analyse or report results for any predictive relationship between AMS severity or occurrence and $S_{pO_{2}}$. Full inclusion and exclusion criteria are listed in Appendix A. Studies involving pharmacological/homeopathic intervention(s) were considered, albeit only included if control/placebo group data could be isolated from those of the treated group(s) and were still relevant in the context of any relationship/difference in AMS.

### 2.3 Information sources

Information sources were identified by searching: (1) PubMed (between database inception and 31 August 2023), PubMed Central (between database inception and 31 August 2023), MEDLINE (between database inception and 31 August 2023), Semantic Scholar (between database inception and 15 June 2023), Cochrane Library (between database inception and 15 June 2023, University of Birmingham Library (between database inception and 15 June 2023) and clinicaltrials.gov databases (between database inception and 31 June 2023); and (2) reference lists of included studies and any relevant literature reviews.

### 2.4 Search strategy

The search strategy used is outlined as follows: [(high‐altitude OR altitude) AND (pulse oximetry OR peripheral oxygen saturation OR peripheral saturation OR peripheral oxygenation OR SpO_2_ OR SaO_2_) AND (acute mountain sickness OR mountain sickness)]. Searches were not limited to the English language, albeit if a full‐length copy could not be obtained for further translation the study was excluded. For key abstracts and unavailable full‐text items, authors were contacted to request further information.

### 2.5 Selection process

Identified sources were managed using Rayyan (Ouzzani et al., 2016). Deduplication was carried out by Rayyan where possible, and any alternative verifiable data duplications further removed. Titles and abstracts were then screened for eligibility using semi‐automated tools in Rayyan, which enabled rapid identification of customised exclusionary terms with manual verification by two reviewers (see Appendix A). If any exclusionary criteria were identified, the study was immediately excluded. If no exclusionary criteria were identified within the title or abstract, and inclusionary criteria were either identified or unclear, the source was advanced to the next screening phase for further evaluation of its eligibility. The next phase aimed to finalise eligibility and consisted of retrieving full‐texts wherever possible and further screening them for any inclusionary/exclusionary criteria. Two reviewers independently reviewed full‐text sources at this time with any disagreements over inclusion/exclusion resolved by an additional reviewer via tie‐breaking. Remaining full‐text sources for included studies were then used to extract data items wherever possible, as outlined next.

### 2.6 Data items

Data items sought included: sample size; oximetry device used; measurement altitude/location, anatomical site (e.g., finger, earlobe), frequency/interval (e.g., every second; every 5 min) and duration (e.g., 90 s), timing (i.e., prospective, on arrival to altitude, at onset of sickness), time of day (e.g., overnight, morning, or post‐exercise), ambient temperature, human state (i.e., awake or asleep), and body position (e.g., supine, seated, standing). Additional variables for which data were sought included: participant characteristics (e.g., male/female, age), rate of ascent (in line with existing guidelines, yes/no), predominant mode of transport (e.g., flight, trekking, or by car), AMS assessment method used (e.g., LLS or ESQ or AMS‐C), prevalence or incidence of AMS, any processing techniques applied to raw oximetry data, and statistical analysis procedures. (Rate of ascent was assessed using the ascent guideline from the Oxford Handbook of Expedition and Wilderness Medicine, which stipulates that above 3000 m, ascent should be no more than 500 m per day with a rest day every 3–4 days.)

### 2.7 Data extraction

At least three independent reviewers extracted data items manually from eligible studies using a standardised data collection form (Appendix B). Discrepancies were resolved by an additional reviewer through discussion.

### 2.8 Risk of bias

Risk of bias was assessed by using a modified Newcastle–Ottawa scoring (NOS) tool (Wells et al., 2014). The NOS was used independently by two reviewers for each included study to examine domains such as selection, compatibility and outcomes/exposure, which were evaluated through a series of questions. Answers to these questions were in alignment with a points/stars‐based system, which translated into one of three quality rankings (i.e., ‘Low’: 3 or 4 stars in the selection domain AND 1 or 2 stars in the comparability domain AND 2 or 3 stars in outcome/exposure domain; ‘Moderate’: 2 stars in the selection domain AND 1 or 2 stars in the comparability domain AND 2 or 3 stars in outcome/exposure domain; or ‘High’: 0 or 1 star in the selection domain OR 0 stars in the comparability domain OR 0 or 1 star in outcome/exposure domain). Any score discrepancies between reviewers were settled by a third independent reviewer. Studies with ‘High’ risk of bias were excluded if there was a sufficient number of other studies where this was not an issue. The ‘Traffic Light’ visualisation tool was used to present results from risk of bias assessments with tabular summary also provided.

### 2.9 Data synthesis/analysis

A flow diagram was used to outline search procedures and detail the number of studies included/excluded at each phase. Graphical and tabular summaries were used to present extracted data such as ascent characteristics, AMS assessment methods, $S_{pO_{2}}$ measurement methods and statistical analysis for included studies. Odds/risk ratios for AMS based on $S_{pO_{2}}$ measured at altitudes >3000 m were originally going to be used for quantitative analysis, but due to the nature of extracted data and lack of uniformity across studies, data synthesis/analysis was predominately qualitative with narrative synthesis outlining the consistencies or inconsistencies between studies for extracted data.

## 3 RESULTS

### 3.1 Search results

**FIGURE 1:**
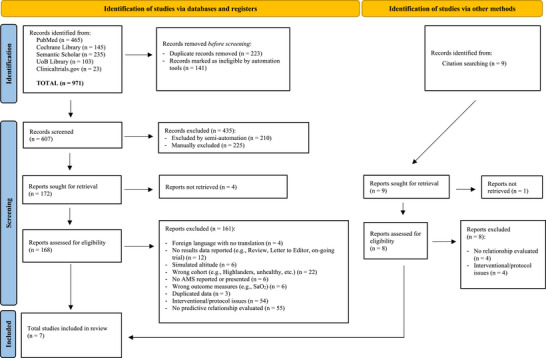
PRISMA diagram for identification, screening and inclusion.

Database search identified 971 sources with nine additional sources identified via reference review. About 223 duplicates were removed along with 141 sources that were marked for ineligibility by automation tools. About 607 abstracts were screened, which resulted in further exclusion of 435 sources (*n* = 210, identification assisted by semi‐automation; and *n* = 225, identified by human). About 168 full‐texts were screened of which 161 were excluded (nine were excluded after tie‐break from 3rd reviewer), with reasons provided in Figure 1, leaving seven studies to ultimately be included.

### 3.2 Extracted data

Results from data extraction for included studies are presented in Tables 1, 2, 3, 4 with key results described in subsections below.

**TABLE 1:** Study characteristics for included studies (*n* = 7).

Author	*n*	M:F ratio	Age range* (years)	Maximum altitude reached (m)	Ascent profiles within guidance	Mode of ascent	AMS assessment method	AMS criteria	Prevalence of AMS
Oliver et al. (2012)	44	1.32	34 ± 13*	5372	Yes	On foot	LLS (Roach et al., 1993)	LLS ≥ 3, with headache and at least one other symptom	AMS‐: *n* = 28 AMS+: *n* = 15 (HAPE: *n* = 1)
Mandolesi et al. (2014)	86 (60)	3.78	37 ± 9.1† 35.7 ± 13.3††	4,559	Mixed	Cable car	LLS (Roach et al., 1993)	LLS ≥ 3 and LLS ≥ 5	AMS+ (LLS ≥ 3): *n* = 24 AMS+ (LLS ≥ 5): *n* = 15
Karinen et al. (2012)	36	17.0	24–45	6300	Mixed	On foot	LLS + clinical score (Hackett & Oelz, 1992)	LLS ≥ 3, with headache and at least one other symptom	No‐AMS: *n* = 12 AMS at 3000 m‐4000 m: *n* = 12 AMS at ≥5000 m: *n* = 12
Chen et al. (2012)	787	1.97	42 ± 11*	3952	Yes	Unclear	LLS (Roach et al., 1993)	LLS ≥ 4, with headache and at least one other symptom, and recent rise in altitude	No‐AMS: *n* = 529 AMS: *n* = 258
Cobb et al. (2021)	332	1.32	43.1 (41.6–44.6)**	5300	Yes	On foot	LLS (Roach et al., 1993)	LLS = 3 or 4 with headache (mild) LLS ≥ 5 with headache (moderate to severe)	No AMS: n = 88 AMS (LLS 3−4): *n* = 77 AMS (LLS ≥ 5): *n* = 167
Karinen et al. (2010)	74	6.40	35 ± 9*	5300	Mixed	On foot	LLS + clinical score (Hackett & Oelz, 1992)	LLS ≥ 3, with headache and at least one other symptom, and recent gain in altitude	AMS+ at: 3500 m, *n* = 8 4300 m, *n* = 17 5300 m, *n* = 27
Modesti et al. (2011)	47	2.13	24–62 (40 ± 9)	5400	Mixed	Cable car	LLS + clinical assessment (Roach et al., 1993)	LLS ≥ 4 with headache	HIGHCARE: 9/15 women and 7/32 men Monte Rosa: 7/17*

Age was presented as range unless otherwise indicated (outlined below). The relative version of the Lake Louise Score (LLS) for acute mountain sickness (AMS) is denoted with parenthetical reference. Rate of ascent was assessed for compliance (Yes/No/Mixed) with ascent guideline from the *Oxford Handbook of Expedition and Wilderness Medicine*, which stipulates that above 3000 m, ascent should be no more than 500 m per day with a rest day every 3–4 days. ‘Mixed’ ascent profiles described studies having multiple ascent profiles with profiles both within this existing guidance, and also at least one profile that violated current guidelines. *Age presented as means ± SD; **Age results presented as means (95% confidence intervals). †Age results presented as means ± SD for the AMS‐positive subgroup. ††Age results presented as means ± SD for AM‐negative subgroup. AMS, acute mountain sickness; AMS+, positive diagnosis of acute mountain sickness; AMS−, negative diagnosis for acute mountain sickness; HAPE, high‐altitude pulmonary oedema.

**TABLE 2:** Extracted data related to $S_{pO_{2}}$ measurements from included studies.

Author	Pulse oximeter used	Anatomical site	Measurement duration	Frequency/interval	Processing	Timing and time of day
Oliver et al. (2012)	Onyx 9500 (Nonin Medical, Plymouth, MN, USA)	Finger	1 min	Lowest and highest values within 1 min period	Average between the high and low values	Daily, morning
Mandolesi et al. (2014)	Pulsox‐300i (Konica Minolta, Tokyo, Japan)	Right index finger	Continuous	Second‐by‐second	Average, and time spent below certain $S_{pO_{2}}$ thresholds	Overnight and during ascent
Karinen et al. (2012)	Onyx 9500 (Nonin Medical, USA)	Finger	2 min	15 s intervals, four times	Average of the four measurements	Morning
Chen et al. (2012)	Oximax N65 (Nellcor Puritan Bennett, Galway, Ireland)	Unspecified	Unspecified	Unspecified	Change/difference in $S_{pO_{2}}$ from 2610 m and 3402 m	Morning at 2610 m and afternoon on arrival 3402 m and next morning
Cobb et al. (2021)	Onyx II 9550 (Nonin Medical, USA)	Unspecified; model used is finger probe	Unspecified	Unspecified	Unspecified	Morning; across multiple expeditions
Karinen et al. (2010)	Onyx 9500 (Nonin Medical, USA)	Finger	2 min	15 s intervals, four times	Average of the four measurements	Unspecified
Modesti et al. (2011)	Life Scope I (Nihon Kohden, Tokyo, Japan)	Unspecified	Unspecified	Unspecified	Unspecified	Unspecified

‘Unspecified’ indicates results not provided in the text. $S_{pO_{2}}$, peripheral oxygen saturation.

**TABLE 3:** Results for extracted data from included studies.

Author	Human state	Body position	Time for physiological stabilisation (yes/no)	Mitigation strategies specified (yes/no)	$S_{{\mathrm{pO}}_{2}}$ compared to $S_{{\mathrm{aO}}_{2}}$ standard (yes/no)	Ambient temperature
Oliver et al. (2012)	At rest	Seated	Yes	Yes	No	−20 to +30°C
Mandolesi et al. (2014)	At rest, sleep and during ascent	Unspecified	N/a	Yes	No	Unspecified
Karinen et al. (2012)	At rest (and post‐exercise)	Seated	Yes	Yes	No	+5 to +20°C
Chen et al. (2012)	At rest	Seated	Yes	No	No	Unspecified
Cobb et al. (2021)	At rest	Seated	Yes	Yes	No	Unspecified
Karinen et al. (2010)	At rest	Seated	Yes	Yes	No	+5 to +20°C
Modesti et al. (2011)	Unspecified	Unspecified	Unspecified	Yes	No	Unspecified

Temperature is presented as a range for ambient temperature measured during measurements unless otherwise specified. Time for physiological stabilisation is indicated as ‘not applicable’ for Mandolesi et al. (2014) as measurements were performed continuously. $S_{{\mathrm{aO}}_{2}}$ is the true oxygen saturation of arterial blood as measured from blood gas sampling, and $S_{pO_{2}}$ is the peripheral oxygen saturation as detected using pulse oximetry. ‘Unspecified’ indicates results not provided in the text.

**TABLE 4:** Statistical analysis methods from data extraction for included studies.

Author	Statistical analysis methods for prediction of acute mountain sickness (AMS)
Oliver et al. (2012)	‐ The following predictor variables were used for longitudinal linear regression analyses using generalised estimation equations (two models): day of expedition, height gain, upper respiratory symptoms, stool consistency, anxiety symptoms, arterial oxygen saturation, heart rate, and fluid intake. ‐ One model analysed all expedition days and the outcome variable was total AMS symptoms. ‐ In the second model, the outcome measure was high altitude headache. ‐ A logistic model (with the same predictors), with the outcome being AMS diagnosis defined by Lake Louise Score, was used to determine whether predictor variables were consistent with being causally related to AMS. ‐ The first two models were rerun using a temporal time‐lag technique. This involved the predictor variables at time point *t* − 1 day being related to the outcome variable of AMS at time point *t* and allowed determination of sequential temporality (i.e., did the predictor variable change before the outcome variable?).
Mandolesi et al. (2014)	‐ Comparison among groups (AMS+ and AMS−) at different altitudes was performed by using repeated‐measures analysis of variance (mixed model). ‐ Pearson's correlation coefficient was used for the single correlation between LLS and $S_{pO_{2}}$ at 3647 m (at rest and all through the night) and during the ascent to 4559 m. ‐ The ROC curve analysis was performed with the statistical software MedCalc (Ostend, Belgium).
Karinen et al. (2012)	‐ Correlations between the LLS and clinical and physiological variables were assessed by Pearson's correlation.
Chen et al. (2012)	‐ To establish a predictive model of AMS development, all patients were randomly classified into a hypothesis‐testing group or validation group (7:3 ratio) by using the random number generator provided with SPSS 18.0 statistical software (SPSS Inc, Chicago, IL, USA). ‐ Statistical comparisons between the hypothesis‐testing group and validation group were performed to confirm that no significant differences existed between the two randomly allocated groups. The hypothesis‐testing group was used to build models, and the validation group was used for model validation. ‐ Multivariate logistic regression analysis using a conditional forward stepwise selection method was performed to analyse the OR of significant parameters associated with subjects who developed AMS. ‐ ROC curve was used in the hypothesis‐testing group to obtain the AUC, sensitivity and specificity.
Cobb et al. (2021)	‐ A univariate logistic regression analysis was performed for each physiological and demographic variable. Variables with *P <* 0.15 were included in a multiple logistic regression analysis for moderate‐to‐severe AMS. ‐ A ROC curve was performed on the predicted probabilities from the logistic regression to assess the goodness of fit.
Karinen et al. (2010)	‐ Spearman's correlations between AMS and $S_{pO_{2}}$. ‐ Sensitivity, specificity and positive and negative predictive values were calculated.
Modesti et al. (2011)	‐ Time patterns of variables measured in the subjects who did and did not experience AMS during the HIGHCARE expedition were compared using a linear mixed‐effect model (group time interaction fitted through the restricted maximum likelihood). Variables independently associated with LLS were then selected through the stepwise multiple linear regression analysis, including data collected during all four steps of the HIGHCARE expedition (baseline, Namche, MEBC1 and MEBC2). In particular, age, sex, haematocrit, body mass index, systolic and diastolic BP, HR, breathing rate, day of expedition, barometric pressure, pulmonary artery pressure, oxygen saturation, catecholamine plasma concentration, and coagulation parameters were included as independent variables with LLS included as dependent variable. ‐ ROC analysis was also performed for oxygen saturation.

Abbreviations: AMS, acute mountain sickness; AUC, area under the curve; BP, blood pressure; HR, heart rate; LLS, Lake Louise Score; MEBC1 or MEBC2, Mount Everest Base Camp 1 or 2; OR, odds ratio; $S_{pO_{2}}$, peripheral oxygen saturation by pulse oximetry; ROC, receiver operating characteristic.

#### 3.2.1 Study characteristics

Populations studied were typically majority male, and middle aged (Table 1). Most studies controlled for confounding health conditions in their study populations with typical exclusionary factors such as cardiovascular disease, recent AMS and pregnancy. The use of medications to assist acclimatisation was controlled for in the inclusion criteria of this systematic review.

#### 3.2.2 Ascent profiles

**FIGURE 2:**
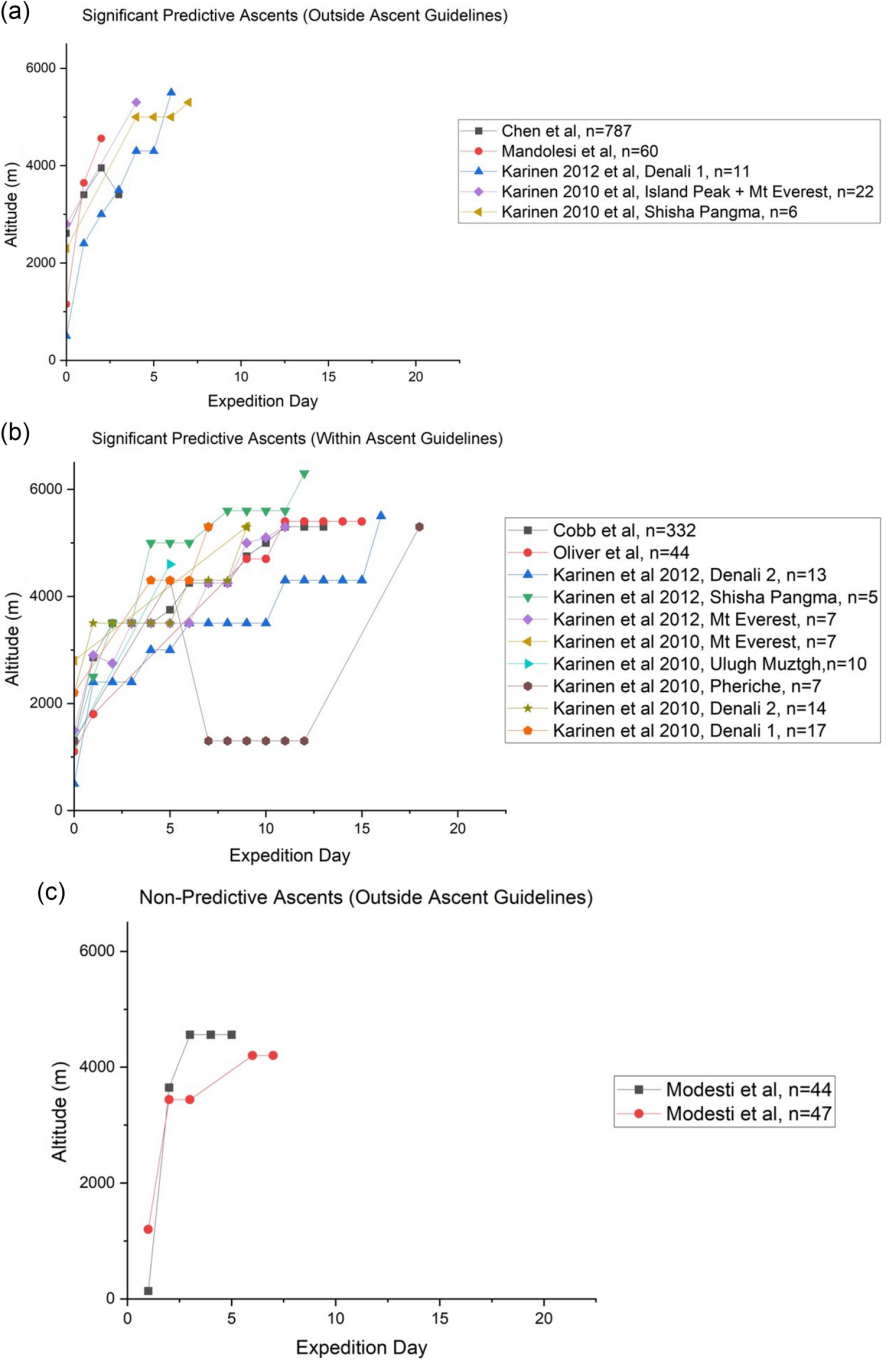
Ascent profiles for included studies. Rate of ascent was considered to be ‘within’ or ‘outside’ guidelines from the *Oxford Handbook of Expedition and Wilderness Medicine*, which stipulates that above 3000 m, ascent should be no more than 500 m per day with a rest day every 3–4 days.

Ascent profiles were reconstructed using data available in the corresponding papers and are illustrated in Figure 2a–c with additional results related to ascent characteristics also provided in Table 1. From Figure 2a–c and Table 1, it is clear that ascent profiles differed greatly between studies with several studies including data from multiple expeditions (having different ascent profiles) over several years for analysis, which were plotted individually in Figure 2a–c (Karinen et al., 2010, 2012; Modesti et al., 2011). Nevertheless, three studies conducted ascents that were in line with existing guidelines related to the maximum rate of ascent at altitude (i.e., increase no more than 500 m per day above 3000 m with rest every 3–4 days) and stayed under 5500 m (Chen et al., 2012; Cobb et al., 2021; Oliver et al., 2012) (refer to ‘Ascent profiles within guidance’ in Table 1). By contrast, studies that included several ascent profiles conducted some that were in line with existing guidance, and others that were not (with one ascent going as high as 6300 m; Karinen et al., 2012) (refer to Table 1).

#### 3.2.3 Mode of ascent

Results from data extracted pertaining to mode of ascent are reported in Table 1. The majority of included studies had climbers ascend by foot. Some studies used a cable car or bus to attain a modest altitude before continuing on foot. Ascent methods were not uniformly reported in detail for every ascent described in the included studies.

#### 3.2.4 Mountain sickness scores

All studies used the LLS for the assessment of AMS, with the LLS version and criteria used by each study highlighted in Table 1. Studies varied in the way that they used the LLS to classify AMS (i.e., AMS‐positive or AMS‐negative). Most studies used LLS ≥ 3 with the presence of a headache for a positive identification of AMS (refer to Table 1), which is consistent with guidance for its use, and also consistent with current guidelines (Roach et al., 1993, 2018). By contrast, two studies used an alternative endpoint for classification, namely LLS  ≥4 with presence of headache (Chen et al., 2012; Modesti et al., 2011). Similarly, Mandolesi and colleagues utilised both LLS  ≥3 and LLS  ≥5 as endpoints for diagnosing AMS and tested $S_{pO_{2}}$ measurements against both endpoints. It should also be noted that Karinen and colleagues (2010, 2012) added the clinical score to LLS.

#### 3.2.5 $S_{pO_{2}}$ measurements

Data extracted in relation to $S_{pO_{2}}$ measurements are reported in Table 2. Studies varied greatly in the degree of information provided surrounding the conduct of pulse oximetry measurements, with many studies providing limited details of their methodology in this area. From the studies that did provide details, a wide variety of protocols were observed. Inconsistencies between studies included but were not limited to: the altitudes at which measurements were taken (between 3952 and 6300 m, refer to Table 1), the time of day (e.g., overnight, morning or post‐exercise), frequency (i.e., continuous or daily each morning), and devices used (refer to Table 2).

Most studies measured $S_{pO_{2}}$ from the finger in the morning using Nonin oximeters (refer to Table 2). Five of the seven included studies collected oximetry measurements while participants were seated at rest, which was preceded by a period of rest (up to 15 min) to allow for physiological stabilisation (Table 3). In addition to resting $S_{pO_{2}}$ measurements, some studies also looked at post‐exercise (or exercise) $S_{pO_{2}}$ (Karinen et al., 2010; Mandolesi et al., 2014). Measurement duration was inconsistent between studies, ranging from continuous to 1–2 min (refer to ‘Measurement duration’ in Table 2), with details surrounding how studies determined values to then be used in subsequent analysis being even less consistent, and often unclear (refer to ‘Processing’ in Table 2).

Despite observed differences, most studies took some action(s) to protect the quality of the $S_{pO_{2}}$ measurements. In addition to allowing for physiological stabilisation, many studies outlined mitigation strategies, which included: participants being sheltered from the wind, wearing gloves and blinded to their results (Oliver et al., 2012); hands covered with mittens (Karinen et al., 2012); no travel to altitudes >2500 m in months prior (i.e., unacclimatised) (Karinen et al., 2010); measurements performed prior to any caffeine consumption (Cobb et al., 2021); and measurements performed in a heated tent (Modesti et al., 2011).

#### 3.2.6 $S_{pO_{2}}$ and relationship with/prediction of AMS

Methods used to evaluate the prediction of AMS by $S_{pO_{2}}$ varied substantially between studies (as outlined in Table 4), producing multiform results (Appendix D), and thus precluding the possibility of carrying out traditional quantitative meta‐analysis. As a result, qualitative meta‐analysis was carried out. Some studies evaluated $S_{pO_{2}}$ as a standalone factor by means of correlation analysis, receiver operator characteristic and logistic regression (Mandolesi et al., 2014; Modesti et al., 2011), while other studies included $S_{pO_{2}}$ as part of multivariate prediction models (refer to Table 4). Studies often included multiple analyses.

Two of the included studies observed that individuals who subsequently developed AMS at higher altitudes had a lower $S_{pO_{2}}$ at lower altitudes than their counterparts who later remained healthy (Cobb et al., 2021; Karinen et al., 2010). Specifically, Karinen et al. (2010) observed this in both resting and exercise $S_{pO_{2}}$ in individuals at 3500 m who later became sick at 4300 m (88 ± 2% vs. 91 ± 3% , *P < *0.05 and 80 ± 2% vs. 85 ± 4%, *P < *0.01, respectively), and between 4300 m and 5300 m (82 ± 4% vs. 86 ± 5%, *P < *0.01, 76 ± 4% vs. 79 ± 5%, *P < *0.01). Cobb and colleagues made similar observations, with individuals who became sick at any point having lower resting and post‐exercise $S_{pO_{2}}$ at 3500 m (88.5% vs. 89.6%, *P = *0.02 and 82.2% vs. 83.8%, *P = *0.027). Similarly, Mandolesi and colleagues observed that individuals who become sick with either mild or moderate‐to‐severe AMS were always more hypoxaemic at rest at altitudes as low as 3275 m (87.7 ± 3.5% vs. 86 ± 4.1% vs. 85.4% ± 4, *P =* 0.037, *P =* 0.030). Further, Chen and colleagues observed the same relationship in resting $S_{pO_{2}}$ at every observation point in their study, even at 2610 m (93.1 ± 2.1% vs. 93.5 ± 2.3%; *P = *0.023), and at 3402 m before and after summiting at 3952 m (86.2 ± 4.7% vs. 87.6 ± 4.3%; *P < *0.001 and 85.5 ± 3.5% vs. 89.6 ± 3.2%; *P < *0.001). Notably, Karinen and colleagues (2012) did not observe a significant relationship between resting $S_{pO_{2}}$ at 2400 m, but found exercise $S_{pO_{2}}$ to be lower at between 3000 and 4300 m in individuals who became sick above 5000 m (*P < *0.05). (Specific data not provided by authors.)

Mandolesi and colleagues derived a cutoff for $S_{pO_{2}}$ of 84% at 3647 m for predicting later development of severe AMS (defined by LLS of >5), which demonstrated 86.67% sensitivity, 82.25% specificity, with area under the receiver operating characteristic curve (AUROC) = 0.87, *P < *0.0001. When restricting analyses to severe AMS defined by LLS ≥ 6, they observed that the sensitivity improved to 90% and the AUROC was 0.91 (*P < *0.0001). These $S_{pO_{2}}$ cutoffs were noticeably lower than the 91.5% $S_{pO_{2}}$ cutoff derived by Modesti and colleagues at 4200 m (sensitivity and specificity only plotted, no values provided). By contrast, Cobb and colleagues did not identify resting $S_{pO_{2}}$ measured at 3500 m to be a standalone predictor (by univariate regression) of severe AMS (LLS ≥ 5) during the trek; however, it was included in the subsequent multivariate regression model based on its significance (i.e., variables with *P < *0.15 considered for inclusion in the multiple logistic regression model; odds ratio (OR) = 0.963 (95% CI: 0.880–1.055)). Nevertheless, Cobb and colleagues did show post‐exercise $S_{pO_{2}}$ to be a significant standalone predictor of severe AMS (OR = 0.870 (95% CI: 0.803–0.943)); however, the AUROC for individual variables was not evaluated.

Some studies examined AMS prediction with $S_{pO_{2}}$ measurements as part of multivariate prediction models. For example, Oliver and colleagues carried out a longitudinal regression analysis and time lag modelling to infer causality of AMS from physiological measurements collected the day before. They identified that $S_{pO_{2}}$ was correlated with high‐altitude headache, but not with AMS. Similarly, Cobb and colleagues included rest and post‐exercise $S_{pO_{2}}$ in their multivariate analysis, which achieved an AUROC of 0.735 (95% CIs: 0.667–0.804, *P < *0.001).

Modesti and colleagues initially identified $S_{pO_{2}}$ as a predictive factor for LLS through stepwise multiple linear regression analysis. However, when measured at 3647 m during a second ascent, $S_{pO_{2}}$ failed validation as an individual predictor (OR = 4.8 (95% CI: 0.5–47.7), *P >* 0.05), and was unable to predict AMS despite being associated with high LLS. Nevertheless, Modesti and colleagues did find that the ‘predictive index’, an algorithm based on the coefficients of several observed predictors (including $S_{pO_{2}}$) exhibited 85% sensitivity and 59% specificity for identifying AMS (OR = 8.1 (95% CI: 1.7–38.6), *P = *0.009).

#### 3.2.7 Other variables linked to AMS prediction

Some included studies revealed predictive relationships between other physiological parameters and subsequent development of AMS. For example, heart rate variability was shown to have a predictive relationship with AMS by Karinen and colleagues (2012). Mandolesi and colleagues noted a significant relationship between heart rate (at rest and overnight at 3647 m) and AMS. Similarly, Oliver and colleagues showed a positive correlation between heart rate and AMS symptom score. By contrast, Karinen and colleagues (2010) did not observe any relationship between resting heart rate at 3500 m and 4300 m and impending AMS at 4300 and 5300 m, respectively.

Modesti and colleagues identified several other factors such as age, sex, body mass index, blood pressure and respiratory rate that were independently associated with AMS (as defined by LLS). Similarly, Modesti and colleagues identified environmental factors including day of expedition and barometric pressure, as well as more complex factors such as coagulation dynamics, haematocrit, pulmonary artery pressure and catecholamine plasma concentration, which were also independent predictors of LLS within multivariate models.

#### 3.2.8 Altitude drugs

Studies that included participants taking medications that enhance acclimatisation were excluded from this study. While it is possible some individuals did not declare use of altitude drugs such as acetazolamide, or were taking prescription medication that invertedly confounded the data, it is not anticipated that any such sporadic drug use occurred at a frequency that would confound the overall study findings, given the total number of individuals included.

### 3.3 Risk of bias

**FIGURE 3:**
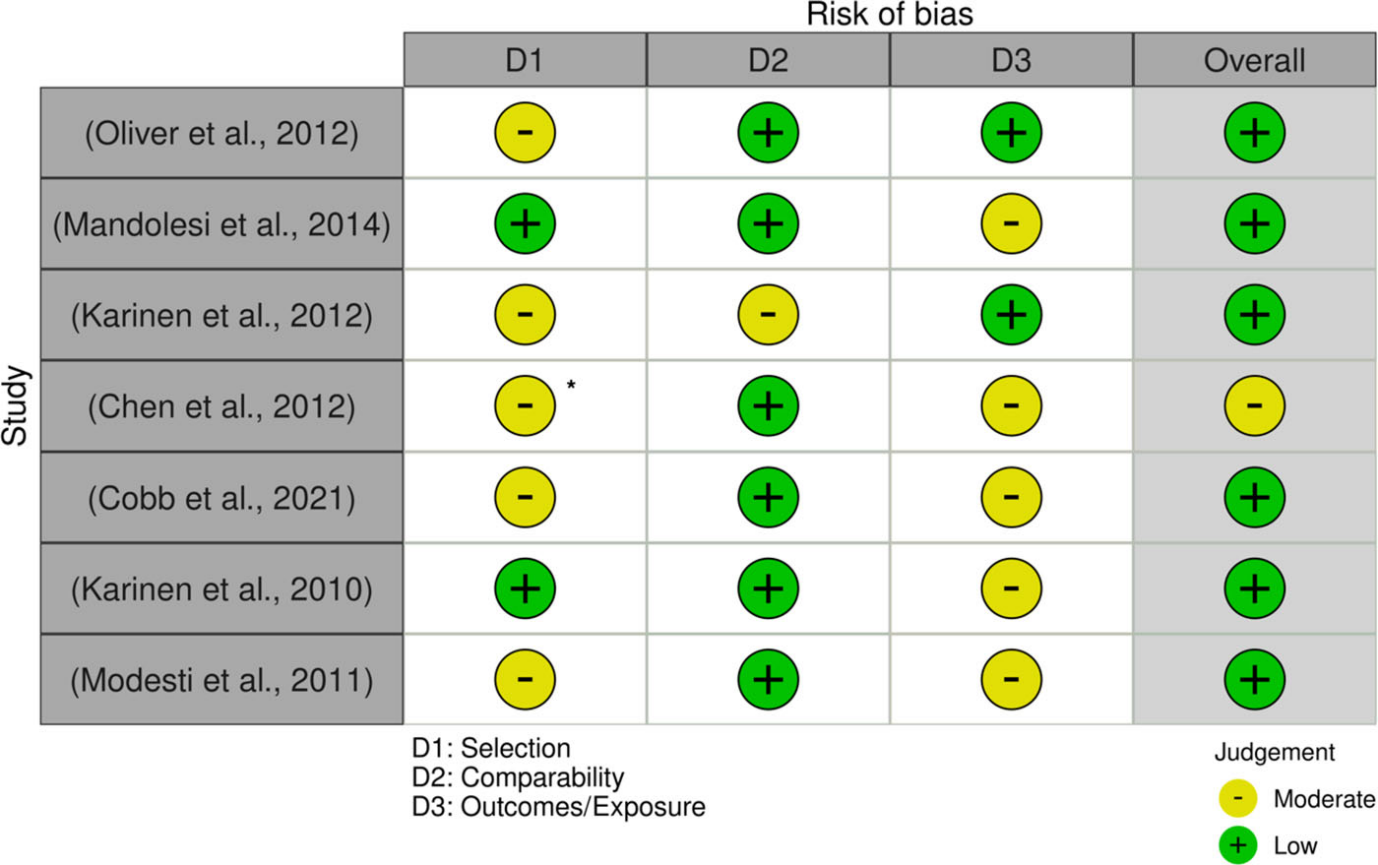
Results from modified Newcastle–Ottawa (NOS) risk of bias assessment plotted using the risk of bias visualisation tool. Scores for each domain were determined by the number of stars awarded. Overall risk of bias was classified based on scores within each domain of the NOS: ‘low’: three or four stars in the selection domain and one or two stars in the comparability domain and two or three stars in outcome/exposure domain; ‘moderate’: two stars in the selection domain and one or two stars in the comparability domain and two or three stars in outcome/exposure domain; or ‘high’: zero or one star in the selection domain or zero stars in the comparability domain or zero or one star in outcome/exposure domain). Asterisk denotes score of two in selection domain (D1) with other ‘moderate’ judgements scoring three in this domain.

Results for the modified Newcastle–Ottawa risk of bias analysis are presented using the ‘Traffic Light’ risk of bias visualisation tool in Figure 3. Risk of bias results demonstrated that all of the included studies had ‘low’ risk of bias except one, which demonstrated ‘moderate’ risk of bias (Chen et al., 2012). Two papers required a third reviewer for tie breaking. After discussion amongst the reviewers, no papers were excluded based on results from the risk of bias assessment.

## 4 DISCUSSION

Our systematic review demonstrates that multiple studies have positively identified a predictive relationship between decreased resting $S_{pO_{2}}$ measured around 3500 to 4000 m and the risk of developing AMS at higher camps. A similar trend between exercising $S_{pO_{2}}$ measurements and AMS was also described by authors of several included studies (Cobb et al., 2021; Karinen et al., 2010, 2012; Mandolesi et al., 2014). There is, however, considerable nuance in the literature surrounding altitude profiles, methodologies, cohorts and measurement techniques, which limited our ability to draw definitive conclusions, as discussed below. This also prevented traditional meta‐analytical techniques being carried out, and required a qualitative review of the evidence. Whilst this may have introduced an element of bias, findings consistent under the scrutiny of different methodological strategies adds an element of robustness to the findings.

Ascents that failed to demonstrate $S_{pO_{2}}$ to be a significant predictive factor for AMS exhibited rates of ascent outside existing recommendations (refer to Figure 2c) (Modesti et al., 2011). This suggests that the predictive relationship may be dependent on rate of ascent. Similarly, the predictive relationship between $S_{pO_{2}}$ and AMS observed in this review (up to 6300 m) may only be relevant up to a threshold point, due to extreme altitude (>5500 m) ascent profiles necessitating extended acclimatisation and partial ascents over extended periods. Unfortunately, none of the predictive models examined in this review adequately addressed ascent profile, which is widely regarded as being one of the most significant risk factors for developing AMS. Thus, caution must be exercised when extrapolating the present findings to such extreme altitudes, or for ascents that go against current recommendations.

There were substantial inconsistencies in $S_{pO_{2}}$ measurement protocols, which posed an important limiting factor. Measurements are susceptible to many confounding factors at altitude including increased UV index and brightness, ambient temperature, and peripheral vasoconstriction due to cold (Luks & Swenson, 2011). Methods for optimising the measurement of $S_{pO_{2}}$ at high altitude do exist (Tannheimer & Lechner, 2019), but these recommendations were not available at the time of publication for six out of the seven included studies. Nevertheless, authors regularly cited strategies used to protect measurement reliability (e.g., sheltering participants from wind and light or having participants wear gloves to warm their fingers) suggesting that authors were aware of the variety of factors that have the potential to influence $S_{pO_{2}}$ readings. Together, this strengthened the findings related to the predictive relationship between $S_{pO_{2}}$ and AMS.

Control for physiological state was carried out with greater consistency across studies than $S_{pO_{2}}$ measurement techniques themselves. Authors often outlined procedures to ensure physiological stabilisation prior to resting measurements (e.g., 15 min of seated rest). However, there were inconsistencies in physiological state (rested vs. during exercise) and the duration of time spent at altitude prior to $S_{pO_{2}}$ measurements (arrival vs. morning after). These variables require adequate control to draw truly robust conclusions such as whether one was more informative than the other regarding prediction of AMS.

Despite positive findings, the utility of $S_{pO_{2}}$ as a standalone predictor was described as limited (Chen et al., 2012). This is most likely due to the substantial overlap in $S_{pO_{2}}$ often observed between groups (AMS and non‐AMS). The use of multivariate analysis methods for prediction models appeared to strengthen the predictive power of $S_{pO_{2}}$ (Cobb et al., 2021; Oliver et al., 2012). Unfortunately, however, multivariate models often included esoteric and difficult to replicate variables such as haematocrit (Modesti et al., 2011), which makes external validation of these models particularly challenging. Nevertheless, multivariate models helped identify other variables that had predictive utility for AMS, particularly that of resting heart rate and heart rate variability, although it is worth noting that not all of the variables identified as having relationships with AMS were found to be individual predictors of AMS. It raises the question as to whether such physiological parameters could be used in combination with $S_{pO_{2}}$ to improve predictive modelling and create clinical tools to identify climbers and trekkers at risk of developing AMS or early identification of AMS

Finally, it must be noted that the variable methods for assessing AMS (i.e., different LLS score cut‐off, and used with and without clinical components) can also impact predictive analysis. Studies included in this review were conducted prior to publication of current guidelines, which omits the sleep component of the LLS (Roach et al., 2018).

### 4.1 Future directions

Future research efforts in this area must focus on the quality and quantity of collected data, ideally with variables that enable easy external validation and re‐validation, a core principle of modelling. The creation of accurate machine learning tools presents a promising option; however, such methods require high‐quality datasets of substantial size, which can be challenging to obtain in the mountain environment. To combat these challenges, future studies should aim to collect data for multiple physiological parameters using high‐fidelity devices (e.g., smartphone‐enabled wearables) across multiple field studies with rates of ascent in line with existing recommendations. Similarly, future researchers must consider the different criteria for LLS published over the years, and must factor this in when conducting any post‐hoc analyses across studies. The addition of the clinical score to the total sum LLS may improve predictions and limit the overall subjectivity of LLS.

### 4.2 Conclusions

In conclusion, this systematic review establishes that there is most likely a predictive relationship between $S_{pO_{2}}$ and AMS. It also highlights that this effect is not profound enough to be of clinical use in isolation. Reviews of existing research, post‐hoc analysis of existing data sets, or the collection of new data could be used to identify other physiological parameters that also share a predictive relationship with AMS.

## AUTHOR CONTRIBUTIONS

Kelsey E. Joyce and Johnathan S.L. Goves—Concept, design, data collection, analysis, and writing; Kimberly Ashdown, Julian Greig, and Sophie Broughton—data collection, analysis, and writing; Arthur R. Bradwell—design and writing; Samuel J.E. Lucas—concept and writing. All authors have read and approved the final version of this manuscript and agree to be accountable for all aspects of the work in ensuring that questions related to the accuracy or integrity of any part of the work are appropriately investigated and resolved. All persons designated as authors qualify for authorship, and all those who qualify for authorship are listed.

## CONFLICT OF INTEREST

Authors have no conflicts of interest to declare.

## Data Availability

Data that support these findings are available upon reasonable request from the corresponding author. Data are not publicly available for privacy reasons.

## APPENDIX A

### Inclusionary and exclusionary criteria. Acute mountain sickness, AMS; and Lake Louise score, LLS

#### Inclusionary criteria were studies that involved

1. Healthy humans (i.e., no pre‐existing cardiac/metabolic/respiratory condition(s), non‐smokers, etc.).
2. Unacclimatised lowlanders, not highlanders/high altitude residents.
3. Collection of peripheral blood oxygen saturation ($S_{pO_{2}}$, via pulse oximetry) at terrestrial high altitude (i.e.,  ≥2100 m; not in environmental chamber or hypoxia delivered via mask).
4. Evaluation of a predictive relationship between $S_{pO_{2}}$ and AMS severity (e.g., LLS).

#### Exclusionary criteria

1. Inclusion of animals.
2. Inclusion of unhealthy humans (e.g., with pre‐existing cardiac/metabolic/respiratory condition(s), smokers).
3. Inclusion of highlanders (living above 2000 m) or acclimatised lowlanders.
4. Utilisation of simulated altitude (e.g., normobaric/hypobaric hypoxia in an environmental chambers).
5. Failure to report AMS assessment method used.
6. Collection of actual or calculated blood oxygen saturation measurements from arterial samples only.
7. Assessment of chronic mountain sickness only.
8. Failure to report statistical method/test and results for any relationship between $S_{pO_{2}}$ and AMS symptomology/severity.
9. Studies measuring $S_{pO_{2}}$ only at a single altitude (e.g., the highest point during ascent) or a maximum altitude less than or equal to 2100 m.
10. Studies involving pharmacological/homeopathic intervention(s) were considered, albeit only included if control/placebo group data could be isolated from that of the treated group(s) and were still relevant in the context of any relationship/difference in AMS.

## APPENDIX B

### Data extraction form. Acute mountain sickness, AMS; Environmental symptoms questionnaire, ESQ; Lake Louise Score, LLS; peripheral oxygen saturation, $S_{pO_{2}}$

#### General

‐ Author and year published.
‐ Article title.
‐ Article type (e.g., review, letter to editor, original research).
‐ Sample size (*n* = ).
‐ Participant demographics (e.g., male/female, age, ethnicity).

#### AMS and ascent

‐ Ascent in accordance with existing with rate of ascent guidance? (Yes/No).
‐ Altitude or range of altitudes across which measurements were collected.
‐ Predominant mode of transport surrounding measurements (e.g., trekking or by car).
‐ AMS assessment method (e.g., LLS or ESQ or AMS‐C) (note: if LLS is used, confirm the year of the criteria used (i.e., Roach et al., 1993 vs. Roach et al., 2018 or ‘Not specified’); similarly, if any additional/modifying criteria were used/applied to AMS assessment methods, these should be noted.
‐ AMS assessment method used in accordance with guidelines? (Yes/No).
‐ Criteria for diagnosing/defining AMS± (e.g., LLS > 3 w/headache or other).

#### Oximetry measurements

‐ Device used.
‐ Anatomical site (e.g., Finger, Earlobe or ‘No information’).
‐ Time for physiological stabilisation before measurement? (Yes/No/No information).
‐ Duration (e.g., 90 s; or No information).
‐ Timing (i.e., prospectively (of illness), on arrival to altitude, at onset of sickness; or No information).
‐ Time of day (e.g., Overnight, Morning or No information).
‐ Frequency/interval (e.g., Every second, Every 5 min or No information).
‐ Human state during measurement (i.e., Awake (at rest), Asleep or No information).
‐ Body position (e.g., Supine, Seated, Standing or No information).
‐ Ambient temperature during measurement (°C / °F or Unspecified).

#### Analysis and results

‐ Processing techniques applied to $S_{pO_{2}}$ data (if any).
‐ Statistical analysis used (e.g., Pearson's *r*, etc.).

## APPENDIX C

### Summary of ethical approval information for included studies

**Table:**

Author	*Declaration of Helsinki*	Ethical approval board	Ethical Ref	Registration	Consent gained?
Oliver et al. (2012)	Not stated	North West Wales Research Ethics Committee Nepal Health Research Council	Not stated	Not stated	Yes, Written
Mandolesi et al. (2014)	Declared compliant	Ethics and Research Committee of the Medical School of the University of Ferrara, Italy	Not stated	Not stated	Yes
Karinen et al. (2012)	Declared compliant	Ethics committee of Tampere University Hospital, Finland	Not stated	Not stated	Yes
Chen et al. (2012)	Not stated	Institutional Review Board at Chang Gung Memorial Hospital, Chang Gung University College of Medicine, Taiwan	Not stated	Not stated.	Yes
Cobb et al. (2021)	Not stated	University College London Research Ethics Committee	Not stated	Not stated	Yes, Written
Karinen et al. (2010)	Declared compliant	Ethics committee of Tampere University Hospital, Finland	Not stated	Not stated	Yes
Modesti et al. (2011)	Not stated	Ethical committee of the University of Milan Bicocca	Not stated	Not stated	Yes, Written

## APPENDIX D

### Main outcomes from included studies

**Table:**

Author	Main outcomes from included studies.
Oliver et al. (2012)	‐ Only upper respiratory symptoms (positive correlation; *P = *0.039, β = 0.119) and $S_{pO_{2}}$ (negative correlation; *P = *0.048, β* = *−0.066) were significant predictors of the presence or absence of clinically defined AMS. ‐ Odds ratios suggested that a 1 unit decrease in $S_{pO_{2}}$ (%) was associated with a 1.068 (1.000−1.141) significantly higher odds of having AMS. ‐ When investigating high‐altitude headache alone, only $S_{pO_{2}}$ was negatively correlated *(P = *0.001, β = −0.017) with the following day's headache severity. ‐ A decrease in $S_{pO_{2}}$ by 5% would increase headache severity the next day by 0.06 units (0.02–0.10).
Mandolesi et al. (2014)	‐ A significant difference between AMS− and AMS+ (LLS > 5 only) was observed for resting $S_{pO_{2}}$ at 1154 m (95.2 ± 1.2% vs. 94.2 ± 1.9%, *P = *0.013). ‐ Resting $S_{pO_{2}}$ at 3275 m was significantly lower than AMS− (87.7 ± 3.5%) in AMS+ (LLS > 3: 86 ± 4.1, *P = *0.037 and LLS > 5: 85.4 ± 4, *P = *0.030). ‐ Resting $S_{pO_{2}}$ on arrival at 3647 m was significantly lower than AMS− (86.6 ± 2.4%) in AMS+ (LLS > 3: 86.6 ± 2.4%, *P = *0.0056 and LLS > 5: 83.8 ± 2%, *P = *0.0098). ‐ Overnight $S_{pO_{2}}$ at 3647 m was significantly lower compared to AMS− (79.2 ± 3.7%) in both AMS+ (LLS > 3; 78 ± 4.6%, *P = *0.016) and AMS+ (LLS > 5; 76.5 ± 3.9%, *P = *0.003). ‐ AMS score in the morning at 3647 m was significantly and inversely correlated with the mean $S_{pO_{2}}$ at rest on the previous afternoon (*r* = −0.32, *P = *0.008). ‐ Significant correlation observed between resting mean overnight $S_{pO_{2}}$ at 3647 m and LLS the following morning (*r* = −0.25, *P = *0.04). ‐ LLS on arrival at 4559 m was significantly and inversely correlated with the mean $S_{pO_{2}}$ during the ascent (*r* = −0.5, *P = *0.003). ‐ For the $S_{pO_{2}}$ cut off value of 84%, ROC analysis (with AMS defined by LLS > 5) demonstrated sensitivity of 86.67% and specificity of 82.25% with an AUC of 0.87 (*P = *0.0001). Restricting the analysis to the subjects exhibiting severe AMS (LLS ≥ 6), sensitivity increased to 90% and the AUC was 0.91 (*P < *0.0001).
Karinen et al. (2012)	‐ There were no significant findings related to resting $S_{pO_{2}}$ at 2400 m and later onset of AMS; however, exercise $S_{pO_{2}}$ was statistically higher in the no‐AMS group compared to the AMS group at 3000–4300 m (95% CI 3 (1−5), *P < *0.01) as well as in the AMS group ≥5000 m group (*P < *0.05). ‐ However, exercise $S_{pO_{2}}$ did not correlate with the AMS altitude (*r* = −0.028).
Chen et al. (2012)	‐ Subjects who developed AMS had significantly lower $S_{pO_{2}}$ than those who did not at: 2610 m (93.1 ± 2.1% vs. 93.5 ± 2.3%; *P = *0.023), on arrival at Paiyun Lodge on day 1 (86.2 ± 4.7% vs. 87.6 ± 4.3%, *P < *0.001), and on the return 3402 m after a summit attempt (85.5 ± 3.5% vs. 89.6 ± 3.2%, *P < *0.001), respectively. ‐ High change in resting $S_{pO_{2}}$ (OR, 1.062; 95% CI 1.023–1.102, *P = *0.001), was significantly associated with the development of AMS. ‐ A change in resting $S_{pO_{2}}$ of 7.29% (AUC = 0.59; 95% CI 0.54–0.65, *P = *0.001) was associated with a sensitivity of 56.59% and 39.47%, and a specificity of 62.97% and 57.86% in the hypothesis‐testing group and the validation groups, respectively.
Cobb et al. (2021)	‐ Resting $S_{pO_{2}}$ (at 3500 m) was significantly lower for AMS+ (scoring > 5; 88.5% (88.0–89.1%)) compared to AMS− 89.6% (89.0–90.3%), *P ≤* 0.05) as was end‐exercise $S_{pO_{2}}$ (AMS−: 83.8% (82.8–84.9%) vs. AMS+ 3–4: 82.2% (81.2 −83.2%) and AMS+ >5: 81.5% (80.9–82.1%), both *P < *0.05). ‐ Resting $S_{pO_{2}}$ (at 5300 m) was significantly lower for AMS+ (scoring 3–4: 76.0% (74.1–78.0%) and scoring > 5: 77.3% (75.9–78.7%); both, *P ≤* 0.05) when compared to AMS− (79.0% (78.4–79.7%)). ‐ Resting $S_{pO_{2}}$ (from 3500 m) was included in the multivariate logistic regression for AMS+ (≥5 LLS). The OR for resting $S_{pO_{2}}$ was 0.963 (0.880−1.055) and the *P*‐value was <0.15 (the cut‐off for being included in the model). ‐ From physiological variables included in the logistic regression model, only exercise $S_{pO_{2}}$ was a significant univariate predictor of AMS+ (0.870 (0.803–0.943), *P ≤* 0.05). ‐ For the logistic regression including multiple variables for AMS+ prediction, the AUC of the ROC was 0.735 (95% CI 0.667–0.804, *P < *0.001).
Karinen et al. (2010)	‐ At 3000 m there was no difference in resting $S_{pO_{2}}$ between AMS− (93 ± 3%) and AMS+ (91 ± 3%) groups. ‐ At 3500 m, resting $S_{pO_{2}}$ was significantly lower in those who went on to develop AMS at 4300 m (88 ± 2; *n* = 17) compared to those who did not (91 ± 3, *P < *0.05; *n* = 66). ‐ At 4300 m, resting $S_{pO_{2}}$ was significantly lower in individuals who went on to develop AMS (*n* = 27) at 5300 m compared to those who did not (*n* = 46; 82 ± 4% vs. 86 ± 5%, respectively, *P < *0.01). ‐ Significant correlations between LLS and resting $S_{pO_{2}}$ were observed at 4300 m (rho = −0.48, *P < *0.05) and 5300 m (rho = −0.48, *P < *0.05). ‐ Resting $S_{pO_{2}}$ cut‐off value of ≤ 85% at 4300 m for the prediction of AMS (LLS ≥ 3) during the trek exhibited 88% sensitivity, 59% specificity, 36% positive predictive value and 95% negative predictive value.
Modesti et al. (2011)	‐ From the HIGHCARE expedition, the stepwise multiple regression of LLS, demonstrated that $S_{pO_{2}}$ significantly decreased the likelihood of high LLS (negative independent association with LLS, β = −0.174, *P < *0.001). ‐ The predictive index measured in Namche had a sensitivity of 76.9% and specificity of 76.5% ‐ to predict AMS at MEBC1 (5400 m) for the cutoff value of 5.92. ROC analysis was also performed for oxygen saturation, haematocrit and cutoff values (<91.5%, <43.5% and >13.5 mm min^−1^, respectively). ‐ In particular, subjects with predictive index ≥5.92 in Namche had an OR of 5.4 (95% CL 1.7−17.4, *P = *0.004) of impending AMS at higher altitude. ‐ Similarly, during a subsequent expedition (Monte Rosa) logistic regression selected the predictive index ≥5.92 (sensitivity 85%, specificity 59%, positive predictive value 71%, negative predictive value 77%) from Gnifetti Hut to be the only predictor of AMS at Margherita Hut (OR = 8.1, 95% CL 1.7 – 38.6, *P = *0.009).

Abbreviations: AUC, area under the curve; CI, confidence interval; CL, confidence level; HIGHCARE, HIGH altitude CArdiovascular Research project at Mount Everest Base Camp; LLS, Lake Louise Score; MEBC1 or MEBC2, Mount Everest Base Camp 1 or 2; OR, odds ratio; ROC, receiver operator characteristic.
